# Impact of multidrug-resistant bacteria on outcome in patients with prolonged weaning

**DOI:** 10.1186/s12890-018-0708-3

**Published:** 2018-08-20

**Authors:** Johannes Bickenbach, Daniel Schöneis, Gernot Marx, Nikolaus Marx, Sebastian Lemmen, Michael Dreher

**Affiliations:** 1 Department of Intensive Care Medicine, Medical Faculty, RWTH Aachen University, Pauwelsstr. 30, D-52074 Aachen, Germany; 2 Department of Cardiology, Pneumology, Angiology and Intensive Care Medicine, Medical Faculty, RWTH Aachen University, Aachen, Germany; 3 Department of Infection Control and Infectious Diseases, Medical Faculty, RWTH Aachen University, Aachen, Germany

**Keywords:** Multidrug resistance, Mechanical ventilator weaning, Bacterial pneumonia, Survival

## Abstract

**Background:**

Pneumonia and septic pneumonic shock are the most common indications for long-term mechanical ventilation and prolonged weaning, independent of any comorbidities. Multidrug resistant (MDR) bacteria are emerging as a cause of pneumonia or occur as a consequence of antimicrobial therapy. The influence of MDR bacteria on outcomes in patients with prolonged weaning is unknown.

**Methods:**

Patients treated in a specialized weaning unit of a university hospital between April 2013 and April 2016 were analyzed. Demographic data, clinical characteristics, length of stay (LOS) in the intensive care unit (ICU) and weaning unit, ventilator-free days and mortality rates were determined in prolonged weaning patients with versus without MDR bacteria (methicillin-resistant *Staphylococcus aureus* bacteria, [MRSA]; extended spectrum beta lactamase [ESBL]- and Gyrase-producing gram negative bacteria resistant to three of four antibiotic groups [3 MRGN]; panresistant *Pseudomonas aeruginosa* and other carbapenemase-producing gram-negative bacteria resistant to all four antibiotic groups [4 MRGN]). Weaning failure was defined as death or discharge with invasive ventilation.

**Results:**

Of 666 patients treated in the weaning unit, 430 fulfilled the inclusion criteria and were included in the analysis. A total of 107 patients had isolates of MDR bacteria suspected as causative pathogens identified during the treatment process. Patients with MDR bacteria had higher SAPS II values at ICU admission and a significantly longer ICU LOS. Four *MRGN P. aeruginosa* and *Acinetobacter baumanii* were the most common MDR bacteria identified. Patients with versus without MDR bacteria had significantly higher arterial carbon dioxide levels at the time of weaning admission and a significantly lower rate of successful weaning (23% vs 31%, *p* < 0.05). Mortality rate on the weaning unit was 12.4% with no difference between the two patient groups. There were no significant differences between patient groups in secondary infections and ventilator-free days.

**Conclusions:**

In patients with pneumonia or septic pneumonic shock undergoing prolonged weaning, infection with MDR bacteria may influence the weaning success rate but does not appear to impact on patient survival.

## Background

Prolonged weaning from mechanical ventilation (MV) with ≥3 spontaneous breathing trials (SBTs) or ≥ 7 days of ventilation defines a group of patients who require complex and protracted treatment to achieve discontinuation of ventilation [1]. Ventilator-associated pneumonia (VAP) and/or pneumonic septic shock are the most common causes of prolonged weaning and are associated with significantly longer length of stay (LOS) in the intensive care unit (ICU) and increased mortality rates [2].

Despite the introduction of care bundles for the prevention of VAP [3], mortality rates still remain high [4] with an incidence of approximately 6 per 1000 ventilator days. In addition, the development of increasing antibiotic resistance, especially among gram-negative (GN) pathogens in VAP, presents a significant challenge in ICU patients. This makes it even more difficult to break the cycle of VAP treatment, prolonged MV and weaning from the ventilator, and improve patient outcomes.

It has previously been shown that the presence of GN, multidrug resistant (MDR) bacteria predicts mortality, pneumonia per se and the complexity of treatment, and that the severity of a critical illness may also be associated with worse outcome [5]. Data from a meta-analysis of 21 studies in patients with MDR versus non-MDR infections showed that the presence of MDR and inadequate treatment of MDR were predictors of mortality [5]. These findings illustrate the clinical relevance of MDR bacteria. However, it is difficult to determine whether it is inappropriate treatment measures that resulted in MDR bacteria or that the MDR bacteria themselves that are the most important factors in contributing to worse outcomes.

Another complicating factor is that patients with prolonged weaning often have important co-morbidities (e.g. chronic obstructive pulmonary disease [COPD], chronic cardiac insufficiency), have received prolonged ICU-based treatment after acute conditions (e.g. septic or cardiogenic shock, acute respiratory failure), and/or have severe weakness of respiratory muscles, fluid overload and recurrent infections. They often require intensive speech and physical therapy, but isolation measures due to the presence of MDR bacteria may influence routine daily care strategies.

Although there are differences between weaning centers due to local circumstances, management and facilities, these centers should all be able to monitor and treat mechanically ventilated patients. The 18-bed weaning unit where this study was conducted is a closed unit that meets all criteria for a fully equipped ICU with respect to patient monitoring, treatment of invasively and non-invasively ventilated patients, and staffing. There is at least one certified doctor in attendance 24 h a day (two assistant doctors and one senior physician during the day and one assistant doctor during the night), nurses (nurse-patient ratio of 1:2), physiotherapists (therapist-patient ratio of 1:6 and a warranted treatment option of 2/day), one speech therapist, and one psychologist.

There is currently a lack of clinical data investigating the effects of MDR bacteria on outcome in patients with prolonged weaning. Several studies have demonstrated an association between MDR bacteria-related VAP and prolonged ICU treatment [6, 7]. However, characteristics of pathogens during the course of treatment and the impact of MDR bacteria on the outcome of prolonged weaning are not known. Therefore, the aim of this study was to assess the prevalence of MDR pathogens and their resistance profile in prolonged weaning patients, and to determine the effects of MDR pathogens on patient outcome.

## Methods

### Study design

This observational study received approval from the Institutional Review Board for Human Studies at the Medical Faculty of the University Hospital Aachen, Germany, and need for informed consent was waived. All analyses were conducted according to the principles of the Declaration of Helsinki.

### Study population

**Fig. 1:**
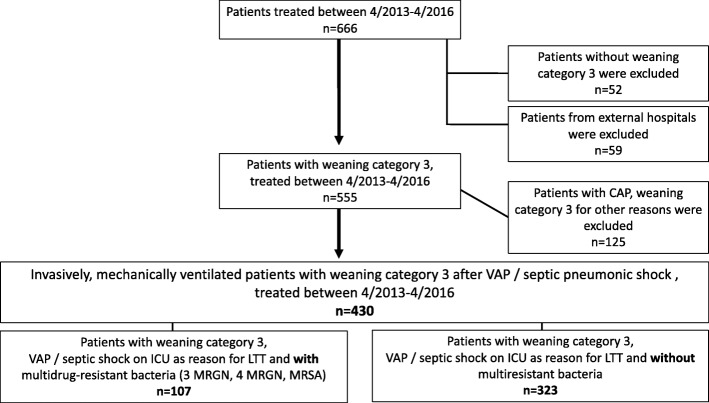
Inclusion and exclusion criteria for the retrospective data analysis. The weaning categories were based on those described previously by Boles et al. [1]. Patients without category 3 (*n* = 52), patients from external hospitals (*n* = 59), and patients with community acquired pneumonia as reason for the initial ICU admission, and patients with other reasons for prolonged weaning were excluded (*n* = 125). CAP, community-acquired pneumonia, VAP, ventilator-associated pneumonia, ICU, intensive care unit, LTT, long-term treatment.

All patients with prolonged weaning treated in the weaning unit over the period April 2013 to April 2016 were eligible. Inclusion criteria were at least one episode of VAP (diagnosed using the Clinical Pulmonary Infection Score [CPIS] [8]) and/or septic pneumonic shock (according to the American College of Chest Physicians [ACCP]/Society of Critical Care Medicine [SCCM] consensus criteria [9]) in the ICU, requiring long-term ventilation, followed by prolonged weaning (≥3 spontaneous breathing trials [SBT] or ≥ 7 days of ventilation) [1]). Patients with community-acquired pneumonia (CAP) and patients with other causes for prolonged weaning, those without prolonged weaning, and patients being admitted from external hospitals were excluded (Fig. 1). In eligible patients, the presence of MDR bacteria was assessed invasively and two groups were defined based on the presence or absence of MDR bacteria.

The primary endpoint was mortality rate on the weaning unit. Secondary endpoints were weaning success rate and ventilator-free days.

### Data collection

Data were retrieved from an electronic patient record system (medico//s, Siemens, Germany) and from an online patient data documentary system (IntelliSpace Critical Care and Anesthesia, ICCA Rev. F.01.01.001, Philips Electronics, the Netherlands). Data on age, sex, pre-existing COPD, pre-existing coronary artery disease (CAD), need for renal replacement therapy (RRT), antibiotic therapy, Simplified Acute Physiology Score (SAPS II) at ICU admission, ICU LOS before admission to the weaning unit, and time on MV before admission to the weaning unit were documented. The following data about treatment in the weaning unit were also extracted: SAPS II at discharge; blood gas analysis on admission; and duration of MV and ventilator-free days. At the time of discharge, patients were classified into the three subgroups of prolonged weaning (weaning category 3) as defined by the German S2 k-guideline [10]:

- 3a: Successful weaning after at least 3 failed SBT or MV longer than 7 days after the first failed SBT without the use of non-invasive ventilation (NIV);

- 3b: Successful weaning after at least 3 failed SBT or MV longer than 7 days after the first failed SBT in combination with NIV; if necessary, continued into out-of-hospital (home) MV;

- 3c: Death or discharge with invasive MV via tracheostomy.

MDR bacteria were categorized into three groups: methicillin-resistant *Staphylococcus aureus* (MRSA); extended spectrum beta lactamase (ESBL)- and Gyrase-producing GN bacteria resistant to three of four antibiotic groups (3 MRGN); and panresistant *Pseudomonas aeruginosa* and other carbapenemase-producing GN bacteria resistant to all four antibiotic groups (4 MRGN) [11, 12].

All MDR bacteria as causative pathogens were isolated from blood probes (and positively identified by at least 1 positive blood culture) or from at least one respiratory specimen (including sputum and tracheobronchial aspirates in MV patients or flexible bronchoscopy with bronchoalveolar lavage [BAL]). Typical contaminants, such as coagulase-negative staphylococci, enterococci or candida spp., were not considered as true pathogens.

### Statistical analysis

Continuous variables were reported as mean values with standard deviations, or medians with interquartile range when data were not distributed normally. Differences between continuous variables were tested using Student’s t-test and Kruskal-Wallis test. Categorical variables were tested using Chi-squared test and McNemar’s test, as appropriate. Ventilator-free days were modelled using a generalized linear model with the logarithmic link function. MDR group was used as the independent variable. In an adjusted model, age (years, continuous), SAPS II (continuous), preexisting pulmonary disease (yes/no), RRT (yes/no) and length of stay (days, continuous) were considered as additional independent variables. Interaction terms were not used. Mortality on the weaning unit was analyzed using separate survival curves for patients in the MDR and non-MDR groups. To compare survival rates in the two patient groups, a Cox proportional hazard model with independent variables MDR group (yes/no), age (years, continuous), SAPS II (continuous), coronary artery disease (CAD) (yes/no), preexisting pulmonary disease (yes/no), and RRT (yes/no) was used. Interactions were not considered. Hazard ratio (HR) and 95% confidence interval (CI) values were estimated.

## Results

A total of 666 patients were treated in the weaning unit over the study period; 430 tracheotomized patients with prolonged weaning from invasive MV met the inclusion criteria and were analyzed (Table 1). There were 107 patients with isolates of MDR bacteria as the causative pathogens for pneumonia/septic shock during the course of treatment. Patients from the MDR group were significantly younger and had a lower incidence of CAD. In addition, MDR versus non-MDR patients had significantly higher SAPS II values at the time of ICU admission (39 ± 9.3 vs. 35.9 ± 8.5, *p* = 0.03) and greater ICU LOS (25.3 ± 17.3 vs. 19.5 ± 12.8, *p* = 0.01). RRT was needed in 25.2% (MDR) and 33.1% (non-MDR) of cases, without no significant difference between the groups.

**Table 1:** Demographic and clinical characteristics of patients with and without multidrug (MDR) bacteria who had prolonged weaning during the intensive care unit stay

Variable	Patients with MDR bacteria (*n* = 107)	Patients without MDR bacteria (*n* = 323)	*p*-value
Age, years	63 ± 15	69 ± 11	< 0.001
Male, n (%)	71 (66.3)	213 (65.9)	0.94
Pre-existing COPD, n (%)	39 (36.4)	98 (30.3)	0.24
Pre-existing CAD, n (%)	28 (26.2)	151 (46.7)	< 0.001
SAPS II at ICU admission	39.0 ± 9.3	35.9 ± 8.5	0.03
Renal replacement therapy during ICU stay, n (%)	27 (25.2)	107 (33.1)	0.127
Days of MV in the ICU	18.1 ± 11.8	17.1 ± 11.4	0.36
ICU LOS, days^a^	25.3 ± 17.3	19.5 ± 12.8	0.01

Data are given as mean ± standard deviation or number of patients (%)

*CAD* Coronary artery disease; *COPD* Chronic obstructive pulmonary disease; *ICU* Intensive care unit; *LOS* Lengh of stay; *MV* Mechanical ventilation; *SAPS II* Simplified Acute Physiology Score II

^a^41 data sets of non-MDR patients missing

**Fig. 2:**
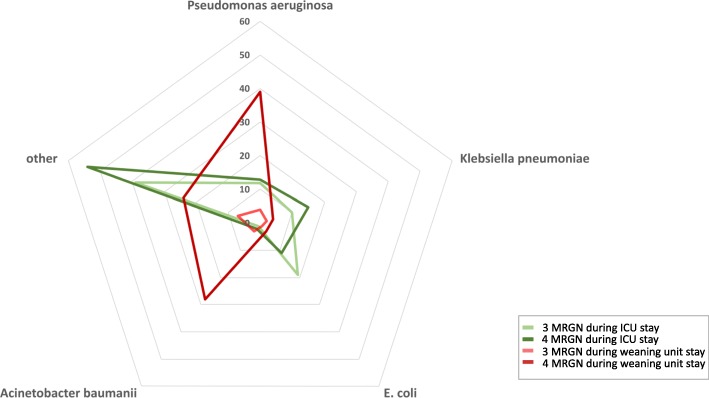
Distribution of gram-negative (GN) multidrug resistant (MDR) bacteria. “Other” pathogens included all other MDR GN bacteria. The scale shows the number of pathogens identified

The proportion of patients with MDR bacteria during ICU and weaning unit stays was 23.8% and 26.9%, respectively. The presence of MRSA in blood culture or respiratory specimens was confirmed in 21 patients during ICU stay (4.8%) and this increased to 39 positive results (9.1%) during time in the weaning unit (*p* = 0.006, McNemar’s test). The chronological distribution of the most relevant 3 MRGN and 4 MRGN strains in the ICU and weaning unit is shown in Fig. 2. In general, there was a marked increase in panresistant bacteria, particularly *P. aeruginosa and Acinetobacter baumanii* during time spent in the weaning unit.

At the time of admission to the weaning unit, arterial carbon dioxide (p_a_CO_2_) was significantly higher in MDR patients (*p* < 0.001) (Table 2); there were no other significant between-group differences in clinical parameters. Recurrent respiratory infection was documented in 37.4% of cases in the MDR group and 43.9% of cases in the non-MDR group.

**Table 2:** Differences between patients with and without multidrug resistant (MDR) bacteria with prolonged weaning during stay in the weaning unit

Variable	Patients with MDR bacteria (*n* = 107)	Patients without MDR bacteria (*n* = 323)	*p*-value
p_a_O_2_ at weaning unit admission, mmHg	87.7 ± 42.2	88.6 ± 27.8	0.83
p_a_CO_2_ at weaning unit admission, mmHg	42.6 ± 9.6	39.6 ± 8.5	< 0.001
pH at weaning unit admission	7.44 ± 0.06	7.44 ± 0.06	0.29
Lactate at weaning unit admission, mmol/L	0.9 ± 0.41	0.9 ± 0.42	0.87
Secondary respiratory infection in the weaning unit^a^, n (%)	40 (37.4)	142 (43.9)	0.28
Days of MV in the weaning unit	15.4 ± 15.8	16.9 ± 22.8	0.31
Weaning unit LOS, days	24.2 ± 26.8	22.9 ± 19.8	0.21
SAPS II at weaning unit discharge	28.3 ± 12.3	29.9 ± 11.5	0.24
Weaning category at discharge^b^			0.05
3a	65 (60.7)	229 (72.0)	
3b	9 (8.4)	13 (4.1)	
3c	33 (30.9)	76 (23.2)	

Data are given as mean ± standard deviation or number of patients (%)

*MV* Mechanical ventilation; *p*_*a*_*CO*_*2*_ Arterial carbon dioxide pressure; *p*_*a*_*O*_*2*_ Arterial oxygen pressure; *SAPS II* Simplified Acute Physiology Score II

Weaning categories were based on the German guidelines for prolonged weaning [10]

^a^Defined as ventilator-associated pneumonia, ventilator-associated tracheobronchitis, pneumonic septic shock

^b^Calculated for *n* = 318, weaning category not defined in 5 data sets of patients without MDR bacteria

Overall mean LOS in the weaning unit was 23.2 ± 21.7 days, with no significant difference between patient groups. Weaning success rates were lower in MDR patients, shown by the smaller proportion of patients in category 3a (defined as patients successfully weaned without any respiratory support) and the higher proportion in category 3c (defined as patients who were in need of invasive home mechanical ventilation or died) compared with the non-MDR group (*p* = 0.05) (Table 2).

**Fig. 3:**
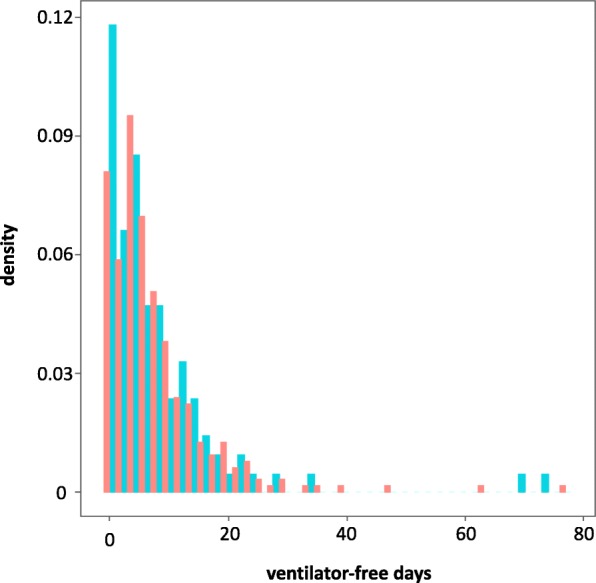
Comparison of the distribution of ventilator-free days in the weaning unit between multidrug resistant (MDR; blue columns) and non-MDR (red columns) patients with prolonged weaning

There was no significant between-group difference in the number of ventilator-free days in patients with and without MDR bacteria. Based on an unadjusted model, there were an estimated 7.2 and 7.4 ventilator-free days in the MDR and non-MDR groups, respectively (the coefficient of multidrug resistance was − 0.03, 95% CI –0.34, 0.24). The distribution of ventilator-free days is shown in Fig. 3.

**Fig. 4:**
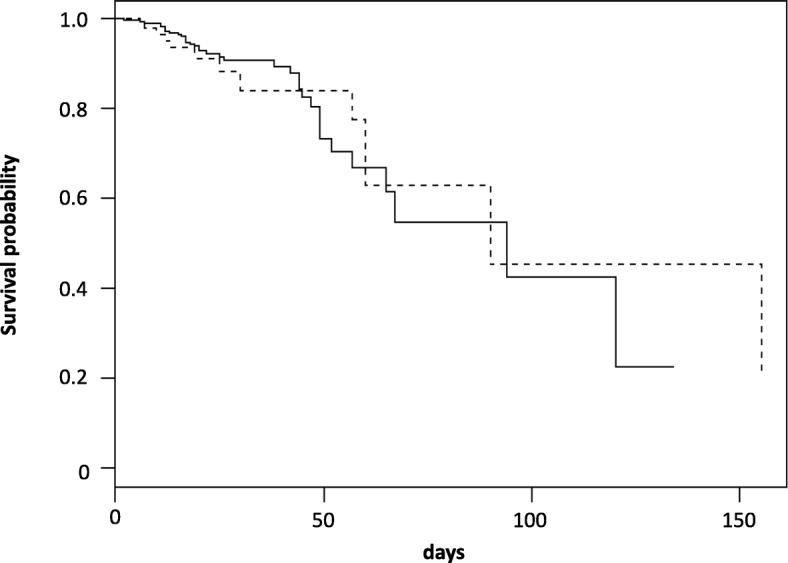
Adjusted Cox model curves for patients with (dashed line) or without (solid line) multidrug resistant (MDR) bacteria. The hazard ratio estimate (0.98, 95% confidence interval 0.49; 1.98) suggests no association between the presence of MDR bacteria and survival

The overall crude mortality rate in the weaning unit for the study population was 12.4%. An adjusted Cox model with consideration of potential coefficents (Table 3) did not show any association between the presence of MDR bacteria and survival, but both age and SAPS were independent predictors of mortality. The survival curve is shown in Fig. 4. In the adjusted Cox model, there were no significant differences in survival between patients with or without MDR bacteria (Fig. 4).

**Table 3:** Summary of analysed coefficents for an adjusted Cox model

Variable	coef	HR	95% CI		*p*-value
Age	0.04	1.04	1.00	1.07	0.03
SAPS II at admission to the weaning unit	0.04	1.04	1.00	1.07	0.03
Pre-existing COPD and/or emphysema	0.10	1.10	0.61	1.98	0.74
Pre-existing CAD	0.13	1.14	0.64	2.03	0.66
Need for renal replacement therapy during the course of treatment	0.13	1.14	0.62	2.09	0.68
MDR bacteria	−0.07	0.93	0.46	1.89	0.84

*CI* Confidence interval; coef, coefficient; *COPD* Chronic obstructive pulmonary disease; *CAD* Coronary artery disease; *HR* Hazard ratio; *MDR* Multidrug resistant; *SAPS II* Simplified Acute Physiology Score II

## Discussion

In this study, approximately one-quarter of infections in patients with prolonged weaning after pneumonia and/or septic pneumonic shock were caused by MDR bacteria, with a marked increase of panresistant bacteria, especially *P. aeruginosa and A. baumanii,* during the course of MV. Although there was no difference in mortality rates and ventilator-free days between patients with an without MDR bacteria, those with infections due to MDR bacteria had higher SAPS II at the time of ICU admission, higher p_a_CO_2_ at the time of admission to the weaning unit, and lower rates of successful prolonged weaning at the time of weaning unit discharge.

VAP is the most common complication in patients needing MV [13] and may further prolonge MV requirement and the weaning process. Our study included a sick group of prolonged weaning patients, as demonstrated by high SAPS II, need for RRT in approximately one-third of patients, and the number of MV days in the ICU before transfer to the weaning unit. This is consistent with previous data on ICU LOS, days on MV and SAPS II in another cohort of long-term mechanically ventilated patients where the most likely reason for prolonged weaning was also the occurence of pneumonia [14].

Our patients with MDR bacteria were significantly younger and had a lower rate of pre-existing CAD compared to those without MDR bacterial infection. However, significantly higher SAPS II suggests that the MDR group was more severely ill during the course of treatment. In addition, significantly higher p_a_CO_2_ in the group with MDR bacteria at the time of weaning unit admission suggest that the weaning process was less advanced, which resulted in a significantly lower rate of complete weaning success at weaning unit discharge. This is in congruence to other studies who could demonstrate that hypercapnia is associated with weaning failure [15] and prolonged weaning, respectively [16]. In our study, one further, potential explanation for higher p_a_CO_2_ is that a high load of MDR bacteria in respiratory specimens could lead to greater secretions or mucus, which would in turn have a negative impact on the weaning process. Another possibility is that co-existing peripheral wounds and skin lesions, which require complex care, might be more extensive in the group with MDR bacteria. These important issues need to be further investigated to facilitate understanding of MDR bacteria-related factors that could influence weaning success rate.

Data on the prevalence of infections with MDR bacteria in patients with prolonged weaning are scarce. In our study, the overall prevalence of MDR bacterial infections was 24.8%, including MRSA, 3 MRGN and 4 MRGN. Increases in MRSA infection rates over time are often seen, most likely due to long-term treatment and repeated courses of antibiotics. A large surveillance study of nearly 150 Spanish ICUs reported that prolonged care and colonisation with several different MDR pathogens were significant, independent risk factors for MRSA colonisation or infection [17]. It is worth noting that MRSA accounted for only 20% of MDR bacteria, and the remaining 80% comprised 3 MRGN and 4 MRGN species. Despite their increasing relevance, there is wide variability in definitions of GN bacteria, making comparison of clinical studies difficult [12]. We particularly noted an increase in 4 MRGN *P. aeruginosa and A. baumanii* in the weaning unit over time, which can be seen both as a result of long-term treatment with inappropriate duration antibiotic therapy and as evidence for increasing bacterial virulence. A sub-analysis of the PNEUMA trial showed that the existence of GN bacteria increased infection recurrence, which is consistent with our findings [18]. In our study, 37.4% of patients with MDR bacterial infection and 43.9% of those not infected with MDR pathogens developed secondary respiratory infections. The between-group difference was not statistically significant, but these high recurrence rates again emphasize the severity of illness in the study population. This makes it difficult to determine whether high mortality rates are attributable to the high pathogenicity of bacteria, inappropriate anitibiotic treatment, or other factors relating to ICU treatment.

In contrast to other studies [19, 20], we did not find any statistically significant difference in mortality rates between patients with or without MDR bacteria. One explanation could be the complexity of patients with prolonged weaning and long-term treatment leading to comparable pathogen-host interactions.

The overall crude mortality rate of 12.4% in our study is similar to that reported by Peñuelas et al. [14], although variations may be due to differences in patient groups (e.g. medical vs. surgical) and institutions [21]. We should point out that our weaning unit is physically separate from the ICU, but has the same monitoring, medical devices and nurse-patient ratio. This enables a systematic, focussed approach to the process of ventilator discontinuation and a procedure of hygiene measures [12] with high adherence to local standards resulting in comparable outcomes in the two patient groups in this study. In addition, the comparability of our unit to an ICU might explain the high illness severity of patients being treated in our weaning unit. Under these conditions, our findings suggest that parameters other than the presence of MDR bacteria appear to have a more dominant influence on survival, as seen in the adjusted Cox model.

Several limitations need to be mentioned when interpreting the results of this study. Firstly, data were obtained retrospectively from a single center. Secondly, the study design only allowed us to examine the distribution of MDR bacteria. These were considered as causative pathogens because they were detected at high concentrations in blood cultures and respiratory specimens. However, it cannot definitively be stated that all MDR pathogens were the source of infection. Finally, we analyzed ventilator-free days and weaning unit mortality, but do not have any data on long-term outcomes in our patients.

## Conclusion

We have shown for the first time the potential impact of infection with MDR bacteria in patients with prolonged weaning. After long-term, complex ICU and weaning unit treatment, mortality rates are quite high but hospital mortality was not affected by the presence of MDR bacteria in our patients. However, MDR bacteria did influence weaning outcome because patients with MDR bacteria had lower rates of successful prolonged weaning. Further prospective studies are needed to analyze infections with MDR bacteria and clinical parameters in this patient group.

## Abbreviations

BAL: Bronchoalveolar lavage
CAP: Community acquired pneumonia
COPD: Chronic obstructive pulmonary disease
CPIS: Clinical pulmonary infection score
GN: Gram negative
ICU: Intensive care unit
LOS: Length of stay
MDR: Multi-drug resistant
MRGN: Multi-resistant gram negative
MV: Mechanical ventilation
RRT: Renal replacement therapy
SAPS: Simplified acute physiology score
SBT: Spontaneous breathing trial
VAP: Ventilator associated pneumonia
WEA: Weaning unit
